# Design and fabrication of a low-cost and programmable dip coating machine

**DOI:** 10.1016/j.ohx.2022.e00364

**Published:** 2022-09-27

**Authors:** Christy Dunlap, Skylar Featherstone, Matthew Smith, Man Vu, Amanda Williams, Jason Bailey, Han Hu

**Affiliations:** Department of Mechanical Engineering, University of Arkansas, Fayetteville, AR 72701, USA

**Keywords:** Dip coating, Precision control, Cycling performance, Electric motor, Arduino, Open-source

## Abstract

Dip coaters provide a compact device that can be used to obtain uniform and consistent coatings on a substrate. This article describes a less costly dip coater design with similar specifications to other dip coaters. The machine consists of a NEMA 23 stepper motor, touch screen display, and acrylic housing to protect the substrate from dust. The machine is programmed using Arduino IDE, allowing for easy changes to be made. The small size of the dip coater makes it ideal for use in labs. The majority of the components require no alterations after purchase, allowing for easy replacements and construction.

## Graphical abstract

**Figure:**
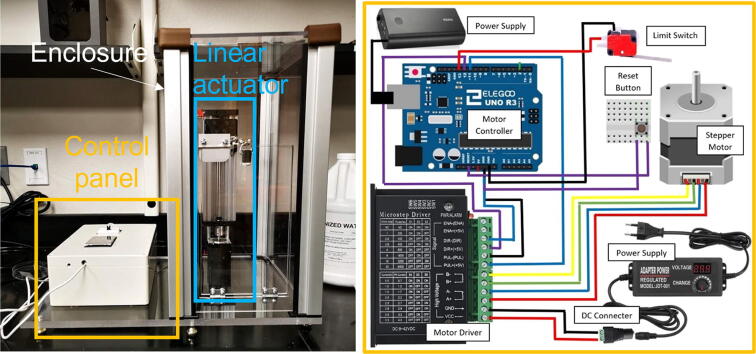

**Specifications table:** 

Hardware name	A Low-Cost and Programmable Dip Coating Machine
Subject area	Engineering and Material Science
Hardware type	Mechanical engineering and materials science
Open Source License	GNU GPL v3
Cost of Hardware	$569
Source File Repository	https://doi.org/10.17605/OSF.IO/3AB6Z

## 1 Hardware in context

A precision dip coater is a machine that holds a substrate and immerses it into a solution, which dwells there motionless to allow the coating to be applied, and then withdraws fully so that it can dry. The immersion and withdrawal occur at constant speeds, controlled by the machine. This process is then repeated depending on the number of cycles required by the customer.

There are several ways to coat a substrate. Some common ways include roll coating, thermal spray, spin coating, and dip coating. Roll coating works by passing a substrate through rollers that are coated. In the thermal spray process as substrate is sprayed with the coating [1]. Spin coating works by distributing a coating by spinning the substrate. Dip coating is the process that dips a substrate directly into a solvent. Each method is useful in different situations. Some are more appealing for factory use while others are better for lab use. There are disadvantages and advantages to each method. For example, spray coating provides a less even coating. By using a dip coater, the user can precisely coat a substrate. The thickness of the coat can be controlled by adjusting how long the substrate is held in the coating and the number of cycles. The amount of the substrate that is covered can be adjusted by changing the travel distance.

Many designs for dip coaters exist in the market. Typically, they include a stepper motor which allows for a very precise and controlled movement; the best sellers include a digital control panel which are programmed to offer adjustable travel distance based on the sample size, control of the speed for both the immersion and withdrawal rate of the sample holder, and control over the number of cycles performed.

A dip coater provides an easy way to coat a substrate to be used in a lab setting. They have been used for a wide range of applications. For example, Mechiakh et al. used a dip coater to deposit TiO_2_ for characterization studies.[2] Mahadik et al. goes over a low cost and generalized method for forming a superhydrophobic coating. [3] Salles et al. tested the effectiveness of Titanium carbide MXene thin films prepared using dip coating as a transparent conductor for use in electrochromic devices. [4] Ma et al. used a developed in-house micro-precision dip coating station to coat microneedles with a molten coating intended for water-insoluble drug delivery [5]. Jeong et al. investigated dip coating film composition with solutions containing small particles [6].

One drawback is the price of precision dip coaters. The most competitive dip coaters on the market range anywhere from $1000 to $3000, yet the price ceiling is much higher [7].

There have been several groups that have designed their own dip coater to avoid spending such high prices. For example, Castillo-Vilcatoma et al. designed a dip coater under 100 dollars using recycled parts. Their machine used an Arduino board and software to achieve a dipping speed range of 0.1–6 mm s^−1^ [8]. Loza M. et al designed a spin coater and dip coater using Arduino [9]. Their dip coating device achieved speeds ranging from 0.6 cm h^−1^ to 30 cm min^−1^. Adámek constructed a dip coater [10]. His devices’ velocity can be set from 0 to 20 mm s^−1^. Mohdisha sought to improve the performance of a dip coater by limiting vibration through their choice in components, such as motor or nuts [11]. These designs are personalized to what components they have available and can be difficult to replicate and adapt or they lack complete instructions for assembly. The dip coater design presented here provides a base model that can be easily modified to meet different specifications.

## 2 Hardware description

The dip coater design is broken into two subassemblies, the machine portion and circuit portion. The machine portion consists of the acrylic panels, linear actuator assembly, and clip. The acrylic panels act as both a base and dust protection layer for the machine. The linear actuator assembly is the main component of the machine, it consists of an acme lead screw and a mount. A clip is attached to the mount through a corner bracket. The clip is used to hold a substrate during dipping. The machine is assembled using nuts and bolts to allow for easy assembly and changes in the design. The top acrylic panel is attached to the rest of the machine through strong Velcro to allow for easy access to the linear actuator and motor. This design also allows for changes to be made to meet the consumer’s needs. For example, if a longer travel distance is desired, then the linear rail guide, acrylic side panels, and quick framing sides would just need to be scaled up. The circuit portion consists of a NEMA 23 stepper motor, ELGOO Uno R3 board (used as a motor controller), a TB6600 motor driver, a limit switch, and a touchscreen shield. The Uno is programmed using Arduino IDE to allow the user to easily input desired specifications such as travel distance, number of cycles, speed, dwelling time, and drying time through the touchscreen [12]. The user also has the option of connecting the ELEGOO Uno R3 to a computer through a USB cord and directly changing the specifications through Arduino IDE. Arduino IDE is commonly used for customizable applications. For example, Lüken et al. uses an Arduino nano microcontroller to control an automated tangential-flow diafiltration device. The controller in their device is used to control pumps, output to a LCD display, and receive information from sensors [13].

The dip coating machine is programmed where when it is initially turned on, a homing button must be pressed to cause the mount to rise until it reaches the limit switch to ensure each use starts from the same location. The design also includes a reset button to stop the machine at any time during use. All of the circuits are housed in a circuit box and mounted to the left of the rest of the machine, allowing for easy access and providing protection for the circuit from the coatings used in the dip coater. This dip coating machine design allows for easy customization from the user and has a broad range of applications.

• Due to the small size of the machine, this dip coater machine is ideal for use in a lab setting.
• It can be used for preparing substrates for studies in material properties.
• The dip coating machine can be used for applying a wide variety of films to a substrate. For example, a test was made with a mesh copper as the substrate and a gel coating was applied with the dip coater to make the mesh superhydrophilic.

## 3 Design files

All the design files (CAD and Arduino code) can be found at https://doi.org/10.17605/OSF.IO/3AB6Z.

### 3.1 Hardware files

**Table:** 

Design file name	File type	Open source license	Location of the file
Complete Assembly	CAD	GPL-3.0	https://osf.io/tk8sn

• See appendix for location of actuator assembly, screws, and additional machine framing CAD files used in the complete assembly (Fig. 18).

#### 3.1.1 Circuit box assembly files

**Table:** 

Design file name	File type	Open source license	Location of the file
Circuits box top	CAD	GPL-3.0	https://osf.io/7txa3
Circuits box bottom	CAD	GPL-3.0	https://osf.io/8qu2s
Riser	CAD	GPL-3.0	https://osf.io/e8bm9
Button	CAD	GPL-3.0	https://osf.io/s2u35
Buttoncover	CAD	GPL-3.0	https://osf.io/xsa2p

• The circuits box top CAD file (Fig. 8) shows what cuts were made in the originally purchased box lid. Holes were made on the top as well as the sides for the touchscreen, reset button, and wires.
• The circuits box bottom CAD file (Fig. 10) shows what holes were drilled in the purchased box to attach it to the acrylic base.
• The riser is a 3d printable piece (Fig. 14) to lift the touchscreen and button to be level with the top of the circuit box.

#### 3.1.2 Machine framing files

**Table:** 

Design file name	File type	Open source license	Location of the file
Door Assembly	CAD	GPL-3.0	https://osf.io/mxnyj
Door Panel	CAD	GPL-3.0	https://osf.io/uw38n
Door	CAD	GPL-3.0	https://osf.io/5erp9
New Housing Base	CAD	GPL-3.0	https://osf.io/rf9ju
Side Panel L&R	CAD	GPL-3.0	https://osf.io/64sef
Side Panel	CAD	GPL-3.0	https://osf.io/bjnux
Top Panel	CAD	GPL-3.0	https://osf.io/b4umj

• The acrylic door panel was purchased to size from tap plastics like the other acrylic sides. However, a door was cut out of this panel from an outside machining shop.
• The acrylic back panel CAD file (Fig. 9) shows the location that holes were drilled in the cut to size acrylic sheet from tap plastic. These holes were added to attach the linear actuator to the machine.
• The machine base CAD file (Fig. 11) shows what holes were drilled in the acrylic panel for the circuit box, leveling feet, and corner quick frame bases to be attached.

### 3.2 Software files

**Table:** 

Design file name	File type	Open source license	Location of the file
dipcoater	Arduino Code	GPL-3.0	https://osf.io/jgtav
Motor_Test_Code	Arduino Code	GPL-3.0	https://osf.io/m64yt

• The Dip Coater Code file has the Arduino code that is uploaded to the ELEGOO Uno R3.
• The Motor Test Code file has Arduino code that can be used to check if the motor is connected properly.

## 4 Bill of materials

### 4.1 Bill of materials - controls components

**Table:** 

Designator	Component	Number	Cost per unit- $	Total Cost - $	Source of materials	Material type
	TB6600 Motor Driver	1	$11.99	$11.99	https://www.amazon.com/UsongShine-Stepper-Controller-Arduino-Printer/dp/B07HHS14VQ/ref=sr_1_1_sspa?dchild=1&keywords=tb6600&qid=1606953122&sr=8-1-spons&psc=1&smid=A1RTFVCI20VZT2&spLa=ZW5jcnlwdGVkUXVhbGlmaWVyPUEyUVk4U1VKMDk5SEsyJmVuY3J5cHRlZElkPUEwOTUzOTE5MlhNVUhOOFhMVkVKQiZlbmNyeXB0ZWRBZElkPUEwNDIwMTUzMUo2NkM5NjhZUUoxRyZ3aWRnZXROYW1lPXNwX2F0ZiZhY3Rpb249Y2xpY2tSZWRpcmVjdCZkb05vdExvZ0NsaWNrPXRydWU=	Non-specific
	ELEGOO UNO *R*3 Starter Kit	1	$29.75	$29.75	https://www.amazon.com/gp/product/B01D8KOZF4/ref=ppx_yo_dt_b_asin_title_o00_s00?ie=UTF8&psc=1	Non-specific
	Excellway Power Supply	1	$10.99	$10.99	https://www.banggood.com/Excellway-9-24V-3A-72W-AC-or-DC-Adapter-Switching-Power-Supply-Regulated-Power-Adapter-Display-EU-Plug-p-1250656.html?akmClientCountry=America&abprots=0&rmmds=search&p=NU271226024945201810&custlixnkid=429307&cur_warehouse=CN	Non-specific
	Ksmile DC Connector Adaptor	1	$5.99	$5.99	https://www.amazon.com/Female-Power-Adapter-Connector-Camera/dp/B01ER6QWAY/ref=sr_1_4?dchild=1&keywords=dc+adaptor+female+connector&qid=1606954404&sr=8-4	Non-specific
	NEMA 23 Stepper Motor	1	$27.99	$27.99	https://openbuildspartstore.com/nema-23-stepper-motor/	Non-specific
	Resistance Touch Screen	1	$18.99	$18.99	https://www.amazon.com/gp/product/B01EUVJYME/ref=ox_sc_act_title_1?smid=A2WWHQ25ENKVJ1&psc=1	Non-specific
	Cylewet 6Pcs V-153-1C25 Micro Limit Switch Long Straight Hinge Lever Arm SPDT Snap Action LOT for Arduino (Pack of 6) CYT1068	1	$6.99	$6.99	https://www.amazon.com/Cylewet-V-153-1C25-Straight-Arduino-CYT1068/dp/B071NSRHK3/ref=sr_1_1_sspa?dchild=1&keywords=limit+switch+for+arduino&qid=1617127748&s=industrial&sr=1-1-spons&psc=1&spLa=ZW5jcnlwdGVkUXVhbGlmaWVyPUE0MzUwNzlSVkM4WkEmZW5jcnlwdGVkSWQ9QTEwMTMyMjM0NUoyWkhVUVBSQVAmZW5jcnlwdGVkQWRJZD1BMDczNTEyOTJGMjdKNlpDMVowVzAmd2lkZ2V0TmFtZT1zcF9hdGYmYWN0aW9uPWNsaWNrUmVkaXJlY3QmZG9Ob3RMb2dDbGljaz10cnVl	Non-specific
	Gifkun Screw Shield Expansion	1	$9.28	$9.28	https://www.amazon.com/Gikfun-Shield-Expansion-Arduino-EK7007/dp/B014SGTP20	Non-specific
	BNTECHGO 18 Gauge Silicone wire 10 ft red and 10 ft black Flexible 18 AWG Stranded Copper Wire	1	$6.98	$6.98	https://www.amazon.com/BNTECHGO-Silicone-Flexible-Strands-Stranded/dp/B01AQOI36M/ref=sr_1_35_sspa?dchild=1&keywords=silicone+wire&qid=1617130186&sr=8-35-spons&psc=1&spLa=ZW5jcnlwdGVkUXVhbGlmaWVyPUEyVk1UUU1GU0cxVzFNJmVuY3J5cHRlZElkPUEwNDQ2NDA5WDJJMFEzWklCRUgzJmVuY3J5cHRlZEFkSWQ9QTEwMzk0OTBHWlpYRTYzRDVaSEcmd2lkZ2V0TmFtZT1zcF9tdGYmYWN0aW9uPWNsaWNrUmVkaXJlY3QmZG9Ob3RMb2dDbGljaz10cnVl	Non-specific

#### 4.2 Bill of materials – Enclosure

**Table:** 

Designator	Component	Number	Cost per unit- $	Total Cost - $	Source of materials	Material type
	Enclosure for Circuits (10.4 × 7.2 × 3.7 in.)	1	$22.99	$22.99	Amazon	Polymer
1	Acrylic Base (Thickness = 1/2 in, Width = 14 in, Length = 20 in, Color: clear)	1	$52.50	$52.50	https://www.tapplastics.com/product/plastics/cut_to_size_plastic/acrylic_sheets_cast_clear/510	Acrylic
2	1.00″ X 1.00″ Tube Profile (Length = 17 in)	4	$4.83	$19.32	https://8020.net/9030.html	Metal
3 (Back panel)10,12 (Door panel)	Acrylic Back and Door Panel (Thickness = 1/4 in, Width = 8 in, Length = 17 in, Color: clear)	2	$13.60	$27.20	https://8020.net/9030.html	Acrylic
4	90 Degree Corner Base Connector	4	$1.45	$5.80	https://8020.net/9242.html	Polymer
5	C-Beam Linear Actuator Bundle	1	$112.99	$112.99	https://openbuildspartstore.com/c-beam-linear-actuator-bundle/	Other
6	Corner Bracket	1	$27.90	$27.90	https://www.mcmaster.com/15655A41/	Metal
7	Miniature Spring Clamp	1	$2.65	$2.65	https://www.mcmaster.com/6076A17/	Metal
9	Acrylic Left and Right Panels (Thickness = 1/4 in, Width = 12 in, Length = 17 in, Color: clear)	2	$20.40	$40.80	https://www.tapplastics.com/product/plastics/cut_to_size_plastic/acrylic_sheets_cast_clear/510	Acrylic
11	Mortise-Mount Hinge with Holes (Nonremovable Pin, Zinc-Plated Steel, 1″ x 1/2″ Door Leaf) (1598A52)	2	$1.12	$2.24	https://www.tapplastics.com/product/plastics/cut_to_size_plastic/acrylic_sheets_cast_clear/510	Metal
13	Draw Latch (Screw on, 304 Stainless Steel, 1–3/4″ Long × 7/8″ Wide) (6082A12)	1	$6.77	$6.77	https://www.mcmaster.com/6082A12/	Metal
14	Quick Frame End Cap	4	$1.25	$5.00	https://8020.net/9210.html	Polymer
15	Acrylic Top Panel (Thickness = 1/4 in, Width = 10 in, Length = 14 in, Color: clear)	1	$14.00	$14.00	https://www.tapplastics.com/product/plastics/cut_to_size_plastic/acrylic_sheets_cast_clear/510	Acrylic
16	Thin Hex Nut - M5	2	$0.10	$0.20	https://openbuildspartstore.com/thin-hex-nut-m5/	Metal
18	Medium-Strength Class 8.8 Steel Hex Head Screw Zinc-Plated, M4 × 0.7 mm Thread, 16 mm Long	1	$7.26	$7.26	https://www.mcmaster.com/91280A136/	Metal
19	Swivel Leveling Mount Zinc-Plated Steel with Rubber Cushion and 10–32 Threaded Hole	6	$4.30	$25.80	https://www.mcmaster.com/6103K81/	Metal
20	Low Profile Screws M5 (10 Pack) (12 mm)	2	$1.09	$2.18	https://openbuildspartstore.com/low-profile-screws-m5-10-pack/	Metal
21	Aluminum Door Stop No. 4483	1	$5.30	$5.30	https://8020.net/4483.html	Metal
26	Hillman #10-32x 1-in Phillips/Slotted Combination-Drive Machine Screws (6-count)	1	$1.28	$1.28	https://www.lowes.com/pd/Hillman-10-32-x-1-in-Phillips-Slotted-Combination-Drive-Machine-Screws-6-Count/3035880	Metal
22 & 23	Hillman #10-32x 3/4-in Phillips/Slotted Combination-Drive Machine Screws (8-count)	1	$1.28	$1.28	https://www.lowes.com/pd/Hillman-10-32-x-3-4-in-Phillips-Slotted-Combination-Drive-Machine-Screws-8-Count/3035879	Metal
24 and 25	Hillman #4-40 × 1/2-in Phillips/Slotted Combination-Drive Machine Screws (14-Count)	1	$1.28	$1.28	https://www.lowes.com/pd/Hillman-4-40-x-1-2-in-Phillips-Slotted-Combination-Drive-Machine-Screws-14-Count/3036645	Metal
	Gorilla Super Glue Gel	1	$6.84	$6.84	https://www.amazon.com/Gorilla-Super-Glue-gram-Clear/dp/B082XGL21J/ref=sr_1_3?dchild=1&keywords=gorilla+glue&qid=1625866263&sr=8-3	

#### 4.3 Bill of materials – Enclosure and controls connections/ finishing touches

**Table:** 

Designator	Component	Number	Cost per unit- $	Total Cost - $	Source of materials	Material type
	15 Series Economy Panel Gasket No. 2115 (30 feet)	16	$0.53	$8.48	https://8020.net/2115.html	Polymer
	Black U Channel Edge Trim Seal EPDM 33/64″ high X 11/32″ Wide − 5 Feet Length (1.55 M)	1	$8.99	$8.99	https://www.amazon.com/dp/B07DGFMZJF/ref=cm_sw_r_cp_api_glt_fabc_5ZT02DGGJVCK1N19714F?_encoding=UTF8&th=1	Polymer
	Alex Tech 10ft-1/4″ 10ft-3/8″ 10ft-1/2″ Split Wire Loom Tubing Wire Conduit – Black	1	$12.99	$12.99	https://www.amazon.com/Alex-Tech-50ft-Tubing-Conduit/dp/B07TWB5MKD/ref=sr_1_3?dchild=1&keywords=electrical%252Btube&qid=1616694105&sr=8-3&th=1	Non-spec
	Stylus Pen	1	$11.87	$11.87	https://www.amazon.com/Stylus-Professional-Navigation-Resistance-Capacitive/dp/B07PCPXP2Z/ref=sr_1_4?dchild=1&keywords=stylus+pen+for+resistance+touchscreen&qid=1624885783&s=electronics&sr=1-4	Non-spec
	Gorilla Super Glue Gel	1	$6.84	$6.84	https://www.amazon.com/Gorilla-Super-Glue-gram-Clear/dp/B082XGL21J/ref=sr_1_3?dchild=1&keywords=gorilla+glue&qid=1625866263&sr=8-3	

The majority of the materials needed can be purchased and used as they come, such as the quick framing, screws, and circuit components. The acrylic front and the circuit lid require holes to be cut out through the use of a laser cutter or saw. The acrylic base, acrylic front, acrylic back, and the bottom of the circuit box require holes to be drilled.

## 5 Build instructions

The construction process is broken down into three separate sections. The first is connecting the controls portion of the device. This includes the connections made in the circuit and uploading the needed code to control the machine. The second section is constructing the enclosure portion of the machine. This includes a linear actuator, acrylic enclosure, and the circuits enclosure. The last section goes over how to connect the machine framing the circuit portion. This includes filling the circuit box, mounting the linear actuator, and covering the wires.

### 5.1 Build instructions - Controls

1. Directly connect the screw shield expansion to the ELEGOO UNO R3 from the starter kit.
2. Connect the touch screen to the screw shield expansion. It will fit directly into the terminals.
3. Connect the TB6600 motor driver to the NEMA 23 Stepper motor. The NEMA 23 motor comes with a 4-conductor wire with an attached connector that plugs directly into the stepper motor. For the TB6600 motor driver, the switches were flipped that correspond to 200 steps per revolution. It is important to note that a motor from a different manufacturer may have a different color set for the wires. For the specified motor, the connections are made as follows:
  a. The yellow wire on stepper motor to B- terminal on the TB6600
  b. The green wire on stepper motor to B+ terminal on the TB6600
  c. Blue wire on stepper motor to A- terminal on the TB6600
  d. The red wire on stepper motor to A+ terminal on the TB6600
4. Connect the DC Connector to the TB6600 motor driver. The connections are made as follows:
  a. GND terminal on the TB6600 to the – terminal on the DC connector using a black wire.
  b. VCC terminal on the TB6600 to the +terminal on the DC connector using a red wire.
5. Plug in the AC/ DC adapter power supply to the DC connector.
  a. The AC/DC adapter power supply is set to 24 V. This power supply is used for just for the motor driver. The ELEGOO UNO R3 Board is powered from the computer.
6. Connect one of the buttons to the small breadboard. Place the button so it is on both sides of the breadboard. Both of these components can be found in the starter kit.
7. Connect two wires to the button. Both are on the same side of the breadboard as shown in Fig. 4.

**Fig. 1:**
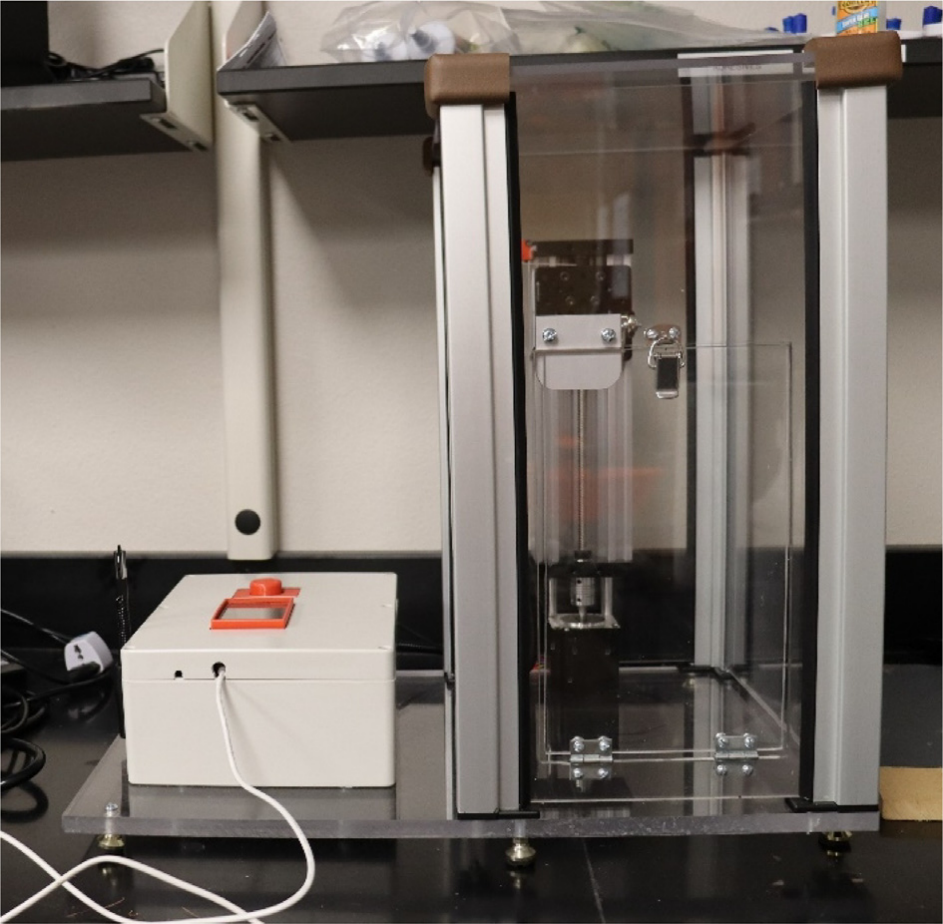
The complete assembly of the dip coating device. The machine consists of a circuits box with a touch screen control pad and an enclosed chamber for the dip coating to take place.

**Fig. 2:**
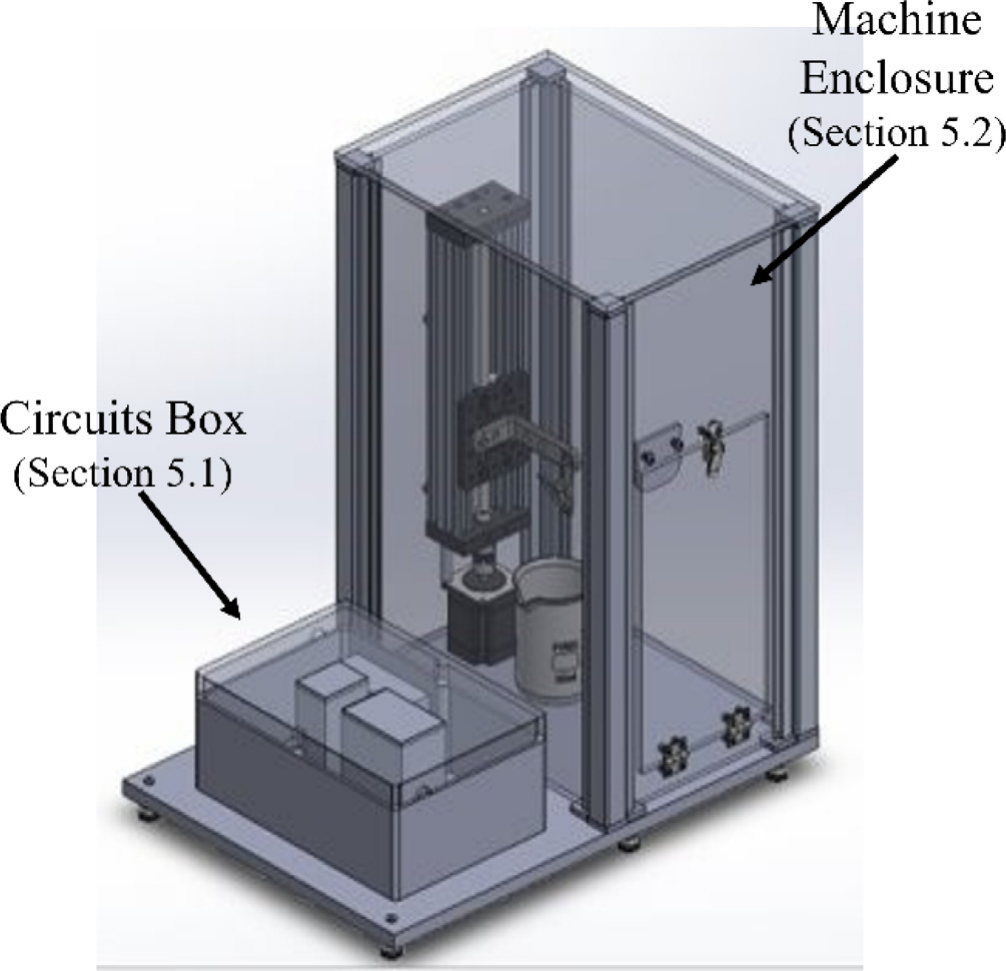
CAD drawing of the design.

**Fig. 3:**
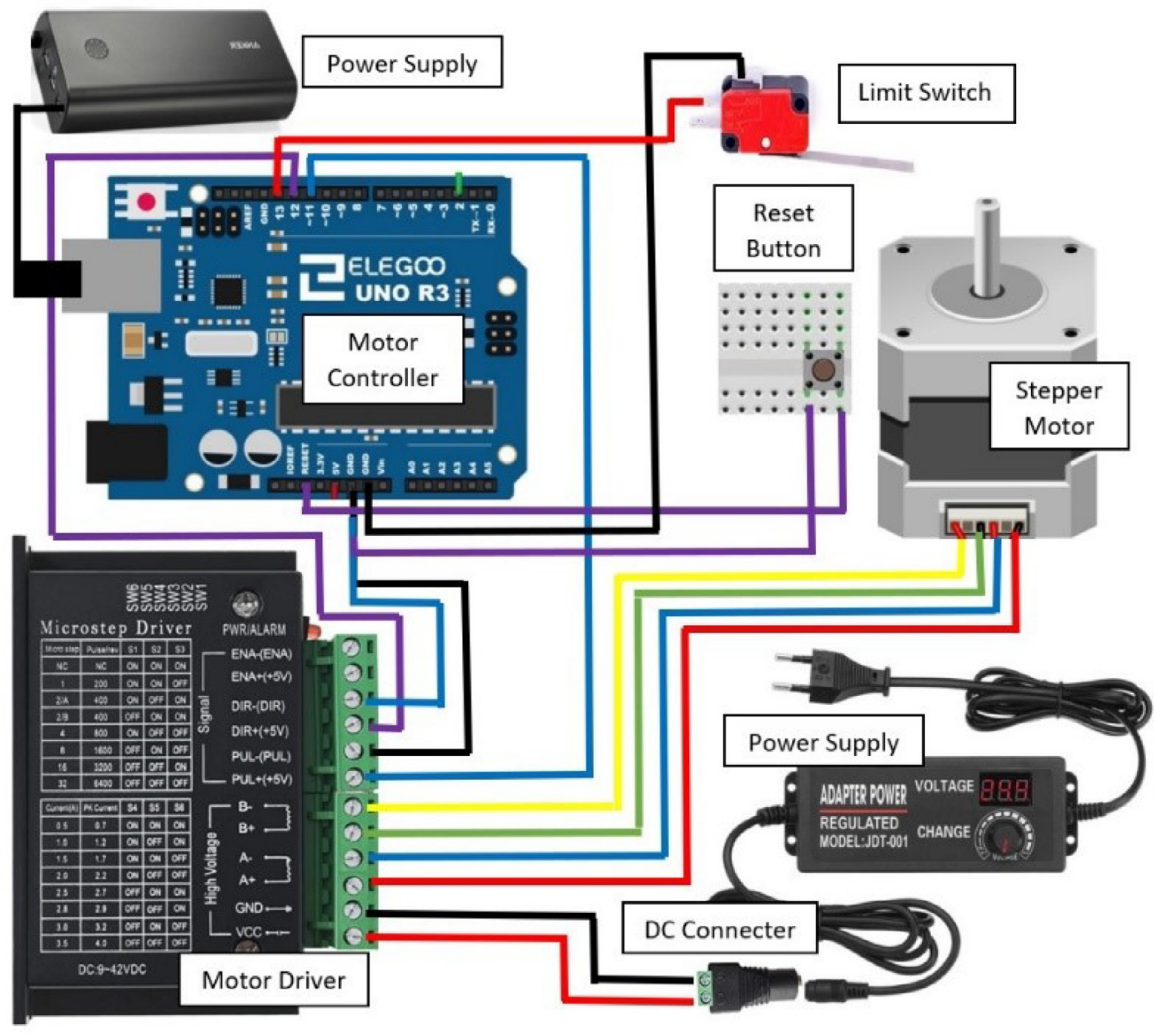
Schematic of circuit excluding the touch screen.

**Fig. 4:**
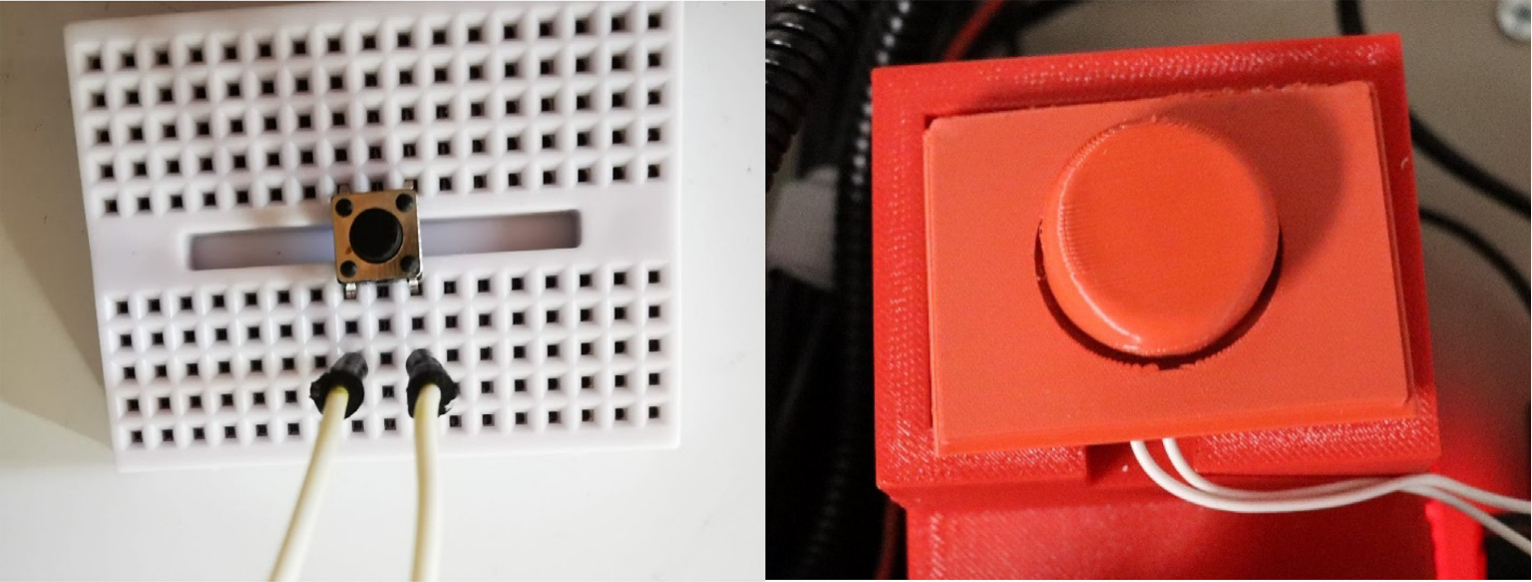
Reset button connected to a small breadboard and covered with 3d printed button cover.
8. Cover button with 3d printed button and button cover. This portion could also be replaced with any button.
9. Solder the red and black wires to the limit switch. Based on the labels in Fig. 5, solder the black wire to Gnd and the red wire to +.

**Fig. 5:**
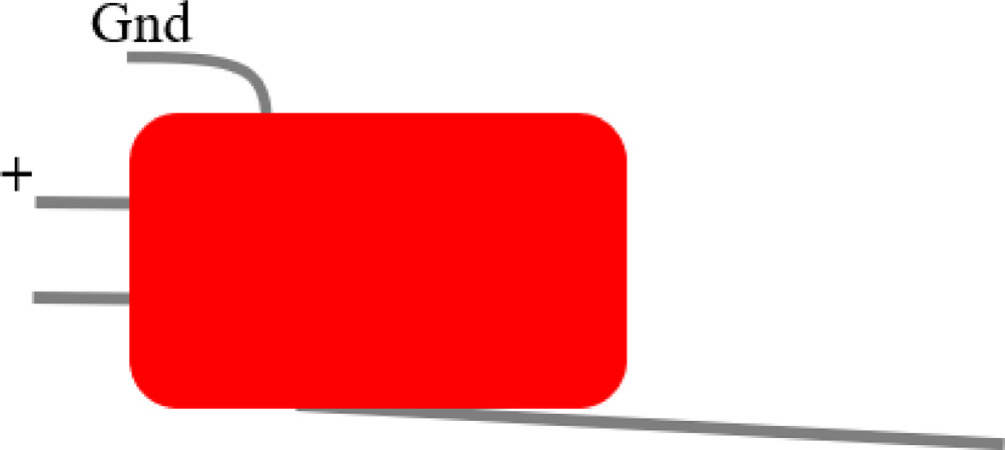
Limit switch with reference labels for connections.
10. Connect all components to the ELEGOO UNO R3. All of the following will be connected through the screw terminals on the screw shield expansion board. Some components will be connected to the same terminal on the ELEGOO UNO R3. Make the connections as follows:
  a. Ground on the ELEGOO UNO R3 to
    i. Gnd on the limit switch (black wire)
    ii. DIR-(DIR) terminal on the TB6600
    iii. PUL- (PUL) terminal on the TB6600
    iv. The button (Either wire connected to the breadboard with the button will work)
  b. Reset pin on the m ELEGOO UNO R3 to the other wire on the breadboard with the button
  c. Pin 13 on the ELEGOO UNO R3 to the + part of the limit switch (red wire)
  d. Pin 12 on the ELEGOO UNO R3 to the DIR+(+5V) terminal on the TB6600
  e. Pin 11 on the ELEGOO UNO R3 to the PUL+(+5V) terminal on the TB6600
11. Use Arduino IDE to open the code provided.
12. Install the needed libraries
  a. Adafruit Touchscreen, Adafruit TFTLCD Library, and Adafruit GFX library
13. Connect the ELEGOO UNO R3 to a computer using the USB connector in the starter kit.
14. In Arduino IDE, open the tools dropdown and chose the board as “Arduino Uno” and select the correct port. Upload the code to the board.
  a. To check to ensure the motor turns the proper way, there is test code included that when uploaded should cause the mount to move down then back up. (Down referring to when the mount is moving toward the motor.)
  b. The other code file has the code needed to run the machine. Once uploaded to the ELEGOO board, the device can be used without being connected to the computer. Fig. 6 presents the flow chart of the code. Although, the device can be used independently, the Arduino code allows for easy editing to be made by the user to meet more specialized specifications needed of the machine. Section 6.2 provides more details on editing the code.

**Fig. 6:**
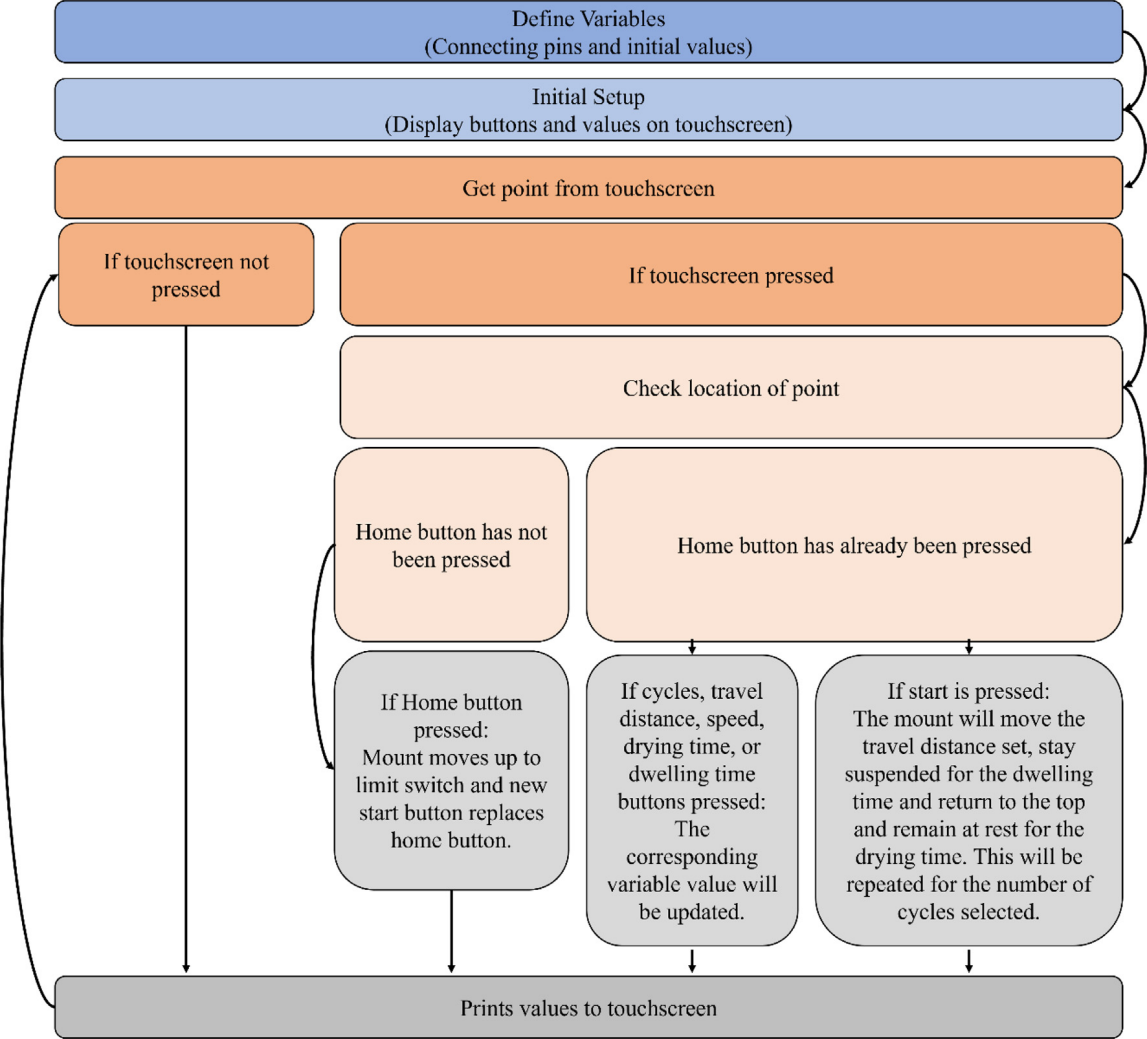
Flow chart for operation procedures of the code.

### 5.2 Building instructions – enclosure

15. Attach the corner bracket (6) to the mount (included in the linear actuator bundle) using a 16 mm long Med-strength class 8.8 steel hex head screw (18) and attach the spring clip (7) to the end of the corner bracket using a Phillips #10-32x 3/4in screw (22,23).
16. Slide 4 12 mm Low Profile Screws M5 (20) screws in the back outermost channels of the linear rail guide from the linear actuator bundle. Two screws on each side.
17. Assemble the linear actuator bundle from open builds and the NEMA 23 stepper motor. There are several tutorials available that can be followed [14]. Be sure to keep the screws in the linear rail guide from step 2 during assembly. The linear actuator bundle includes a lead screw with a 2 mm pitch. The assembled linear actuator with the motor, corner bracket, and clip are shown in Fig. 7.

**Fig. 7:**
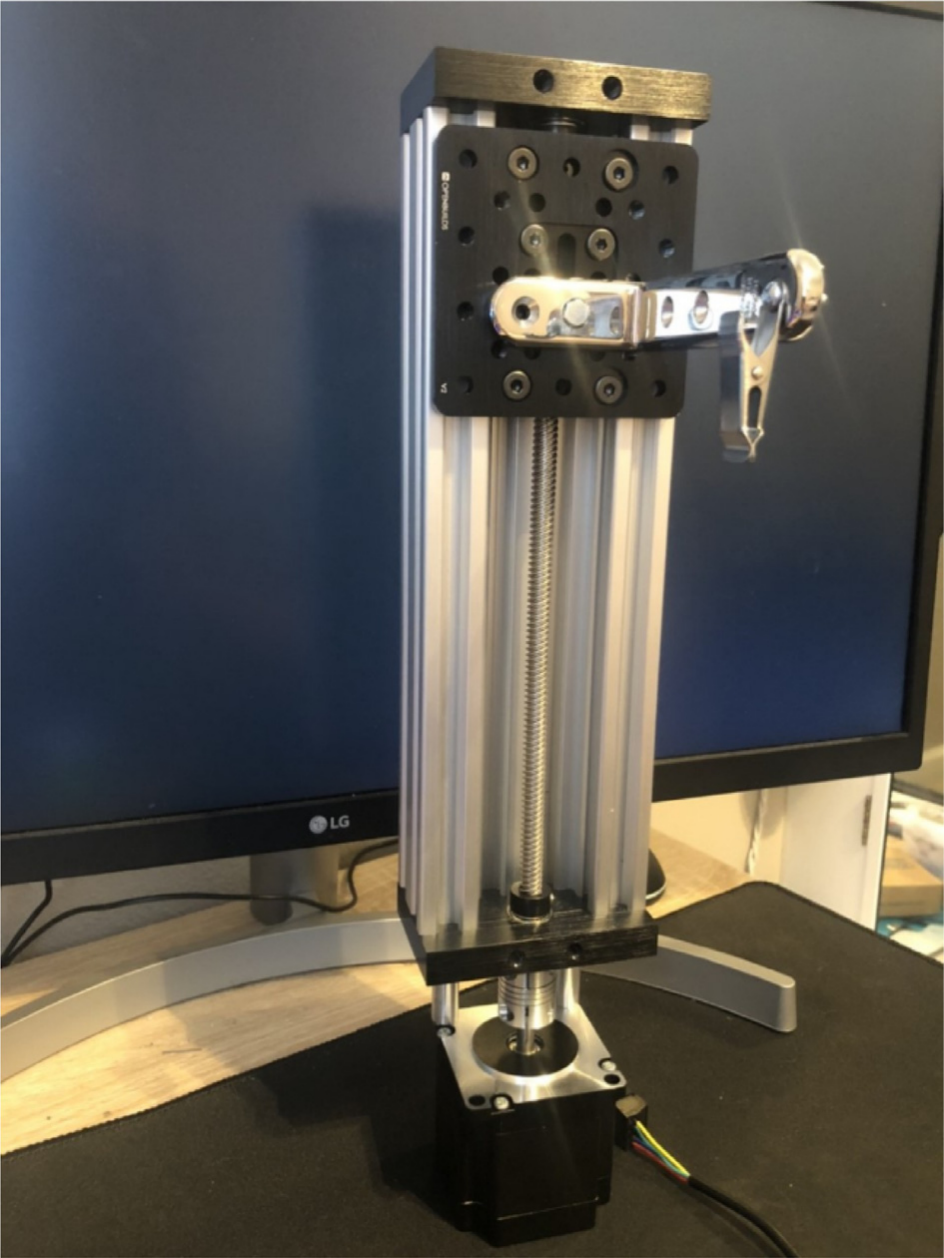
Assembled linear actuator bundle.
18. Have the door cut out of front acrylic piece (10,12) as shown in Fig. 8 by taking acrylic to a machine shop. Both pieces will be used in the design.

**Fig. 8:**
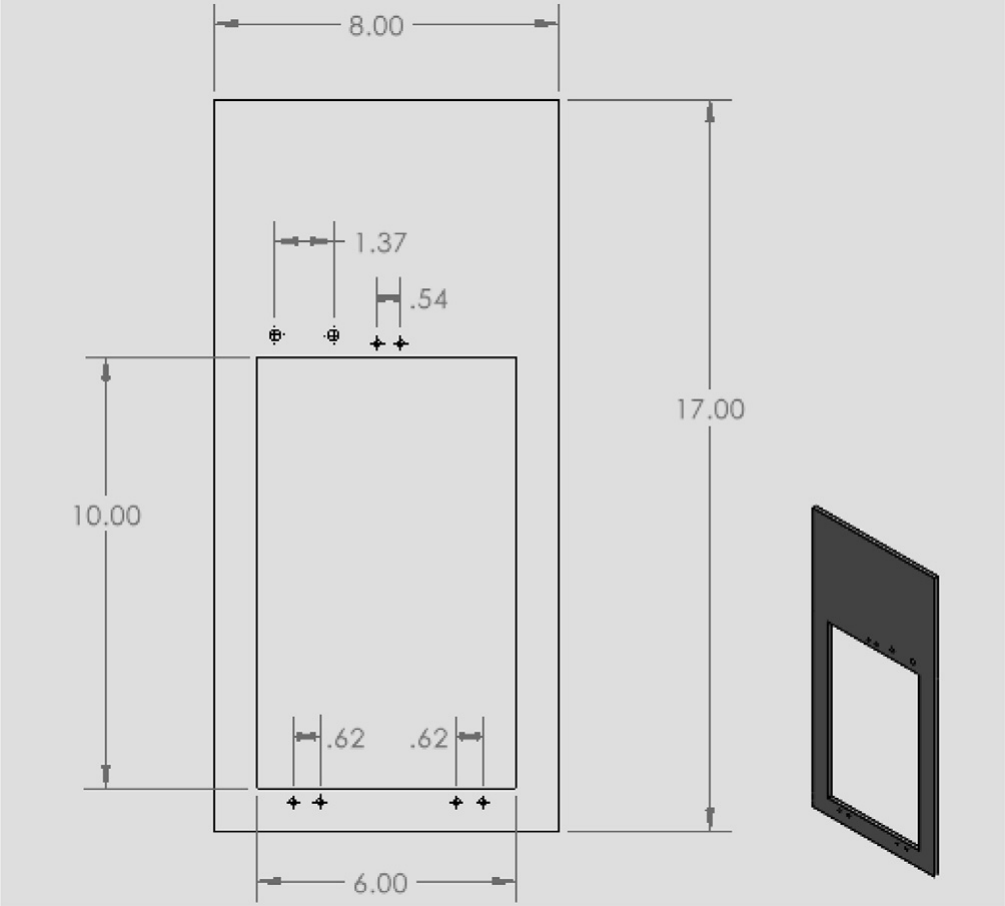
CAD drawing of the acrylic door panel.

**Fig. 9:**
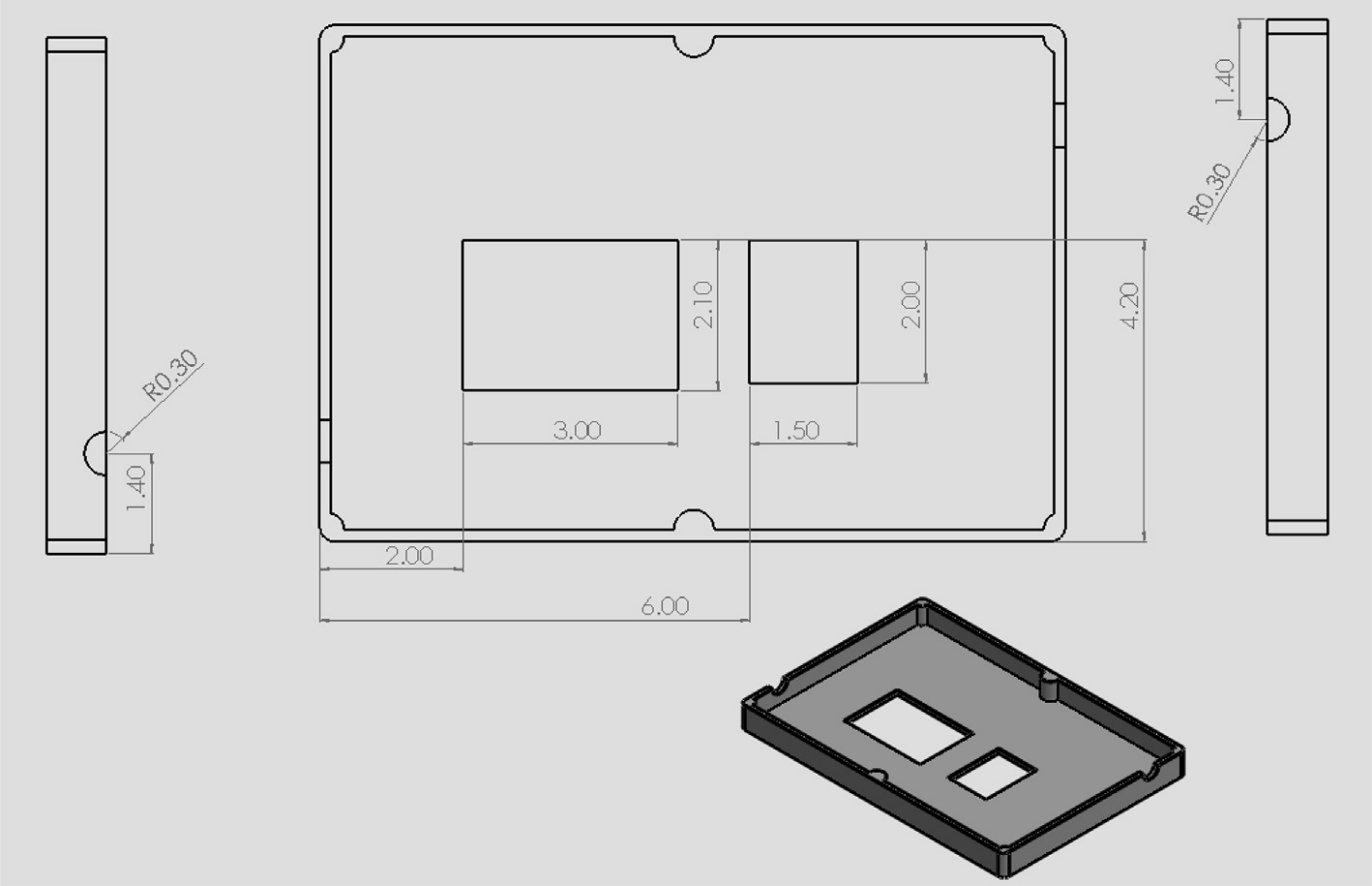
CAD drawing of circuit box lid showing holes that were made for the touchscreen, reset button, and wires. (Drawing dimensions are in inches).

**Fig. 10:**
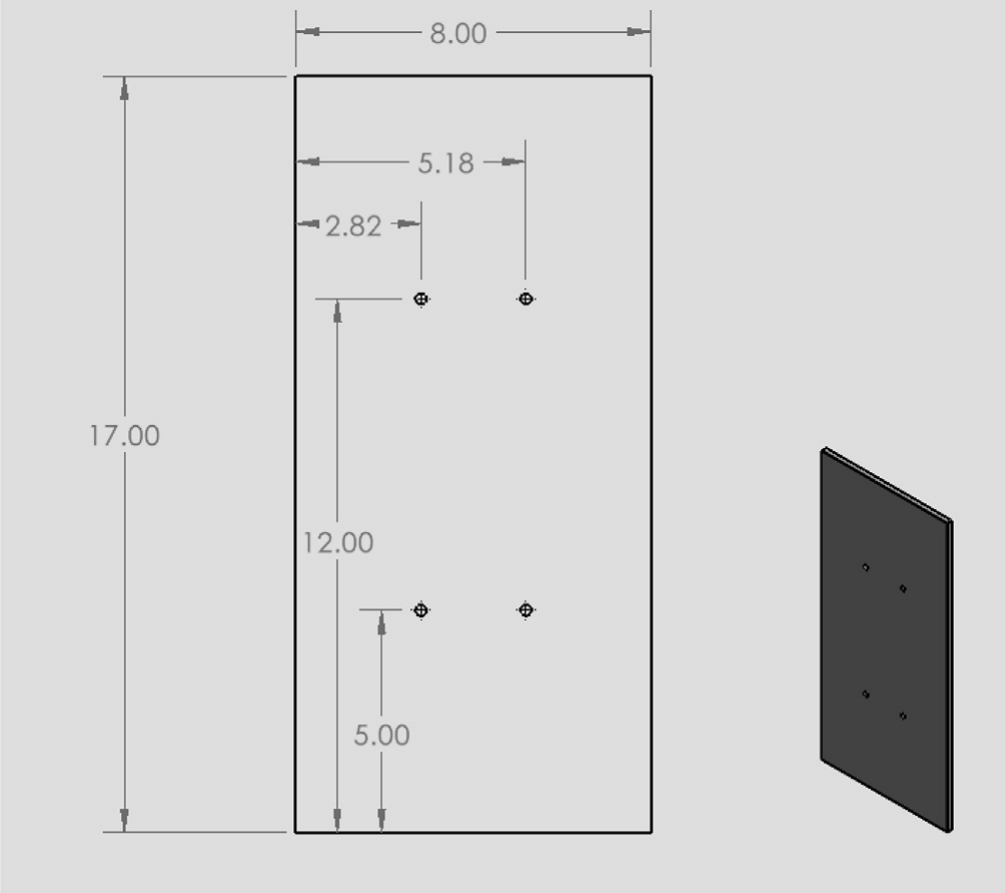
CAD drawing of the acrylic back. The holes are drilled to attach the linear actuator.

**Fig. 11:**
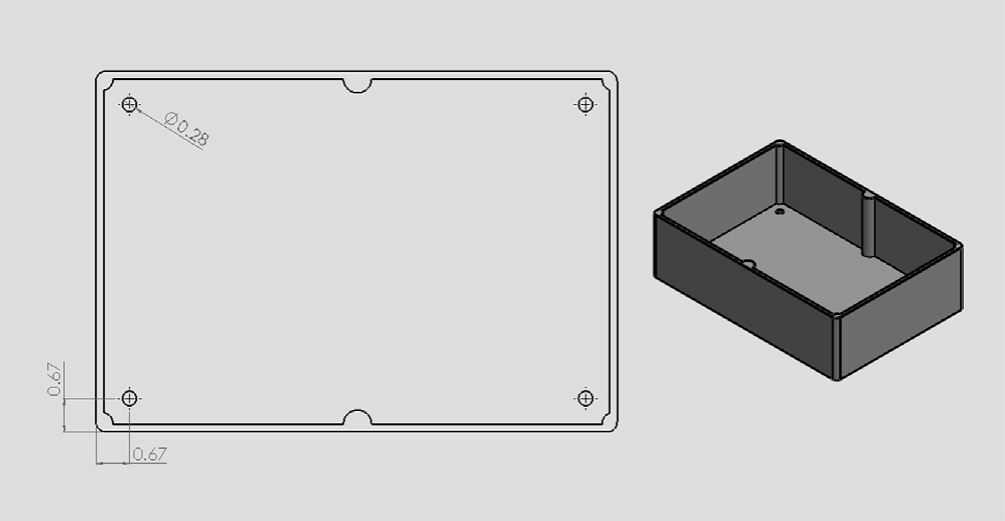
CAD drawing of circuit box bottom showing 4 holes made to attach the box to the machine base. (Drawing dimensions are in inches).
19. Have holes in the circuit enclosure lid cut out as shown in Fig. 9. These holes are for the touchscreen and reset button.
20. Drill 8 holes in the front acrylic piece (10) for the hinges, latch, and doorstop and 8 holes in the door portion of the front panel (12) for hinges and the bottom part of the latch.
21. Drill 4 holes in the back acrylic piece to be used for attaching the linear actuator as shown in Fig. 10.
22. Drill 4 holes in the circuit enclosure base as shown in Fig. 11 for mounting the circuit box to the base of the machine.
23. Drill holes in the acrylic base (1) shown in Fig. 12. These are holes for attaching the quick framing base connectors, circuit box, and swiveling level mounts.

**Fig. 12:**
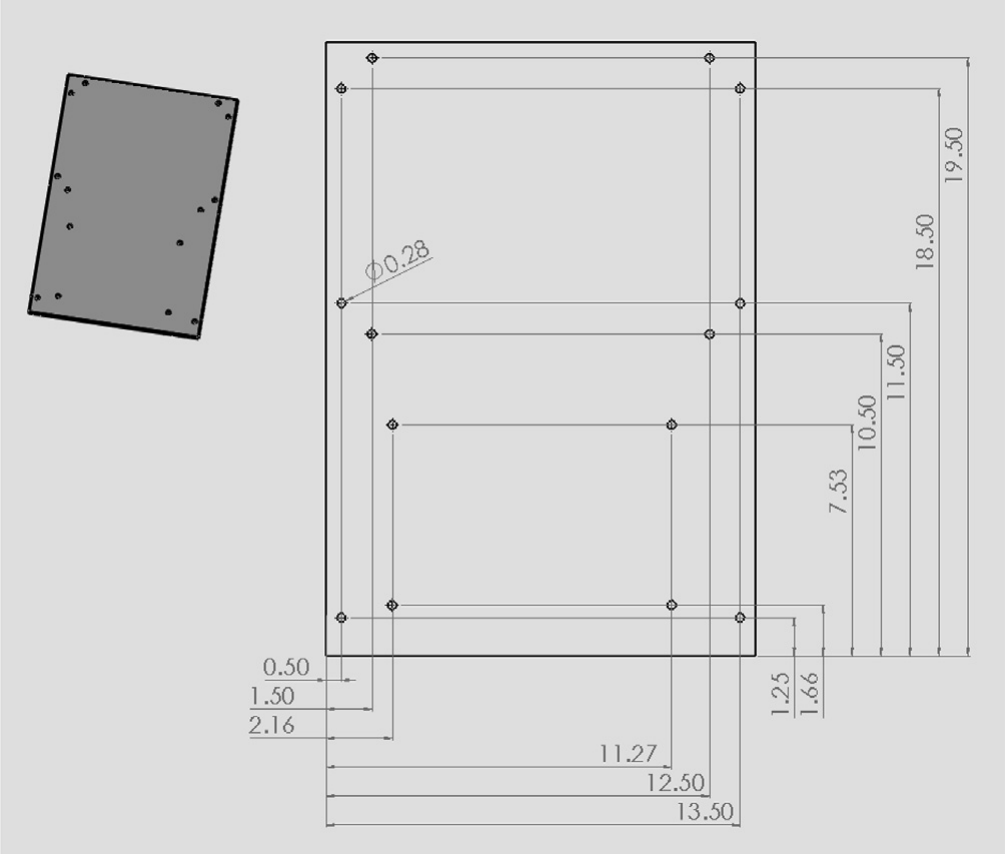
CAD drawing of the acrylic base showing what holes were made to attach the circuit box and machine enclosure. (Drawing dimensions are in inches).
24. Attach hinges (11) and latch (13) using size 4, ½ in long Phillips screws (24 and 25) and attach the doorstop using size 10, ¾ in long Phillips screws (22 and 23) to the acrylic front panel and door (10,12).
25. Attach 90-degree corner base connectors (4), circuit box, and leveling mounts (19) to acrylic base (1) shown in Fig. 13. All are attached using size 10, 1 in long Phillips screws (26). For each 90-degree corner base connector, one side is held with a hex nut while the other side is screwed directly into one of the swiveling level mounts.

**Fig. 13:**
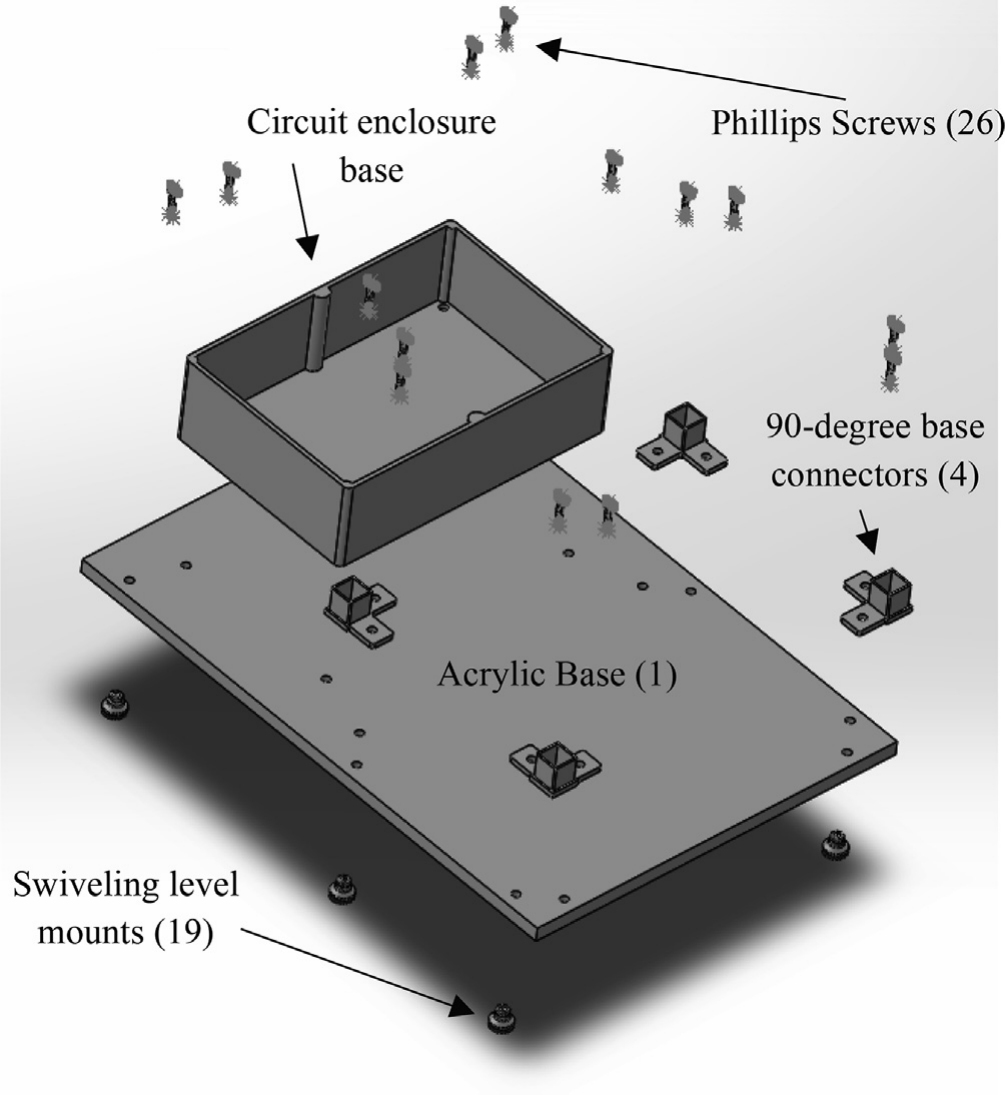
CAD drawing showing how the circuit box, 90-degree corner base connectors, and leveling mounts should be attached to the acrylic base.
26. Slide the quick framing sides (2) over each of the base connectors (4) shown in Fig. 14.
27. Put the gasket on the front and back of both sides of the side acrylic panels (9) and slide them into the grooves of the quick framing on the right and left sides of the machine.
28. Put the gasket on the front and back of both sides of the front acrylic panel (10,12) and slide into the grooves of the quick framing in the front of the machine.

### 5.3 Building instructions – connecting the circuit to the Machine

29. Use glue to attach the limit switch to the top of the linear actuator.
30. Place the linear actuator and motor in the machine.
31. Use the wire loom tubing to cover the wires from the limit switch and motor.
32. Put the gasket on the front and back of both sides of the back acrylic panel (3) and slide into the grooves of the quick framing on the back.
33. Line up the screws on the back of the linear rail guide with the holes in the acrylic back panel (3).
34. Use 4 M5 hex nuts (16) to secure the linear rail guide to the acrylic back panel.
35. Either 3D print the riser block shown in Fig. 15 or use risers to lift the touch screen and reset button to the height of the box.

**Fig. 14:**
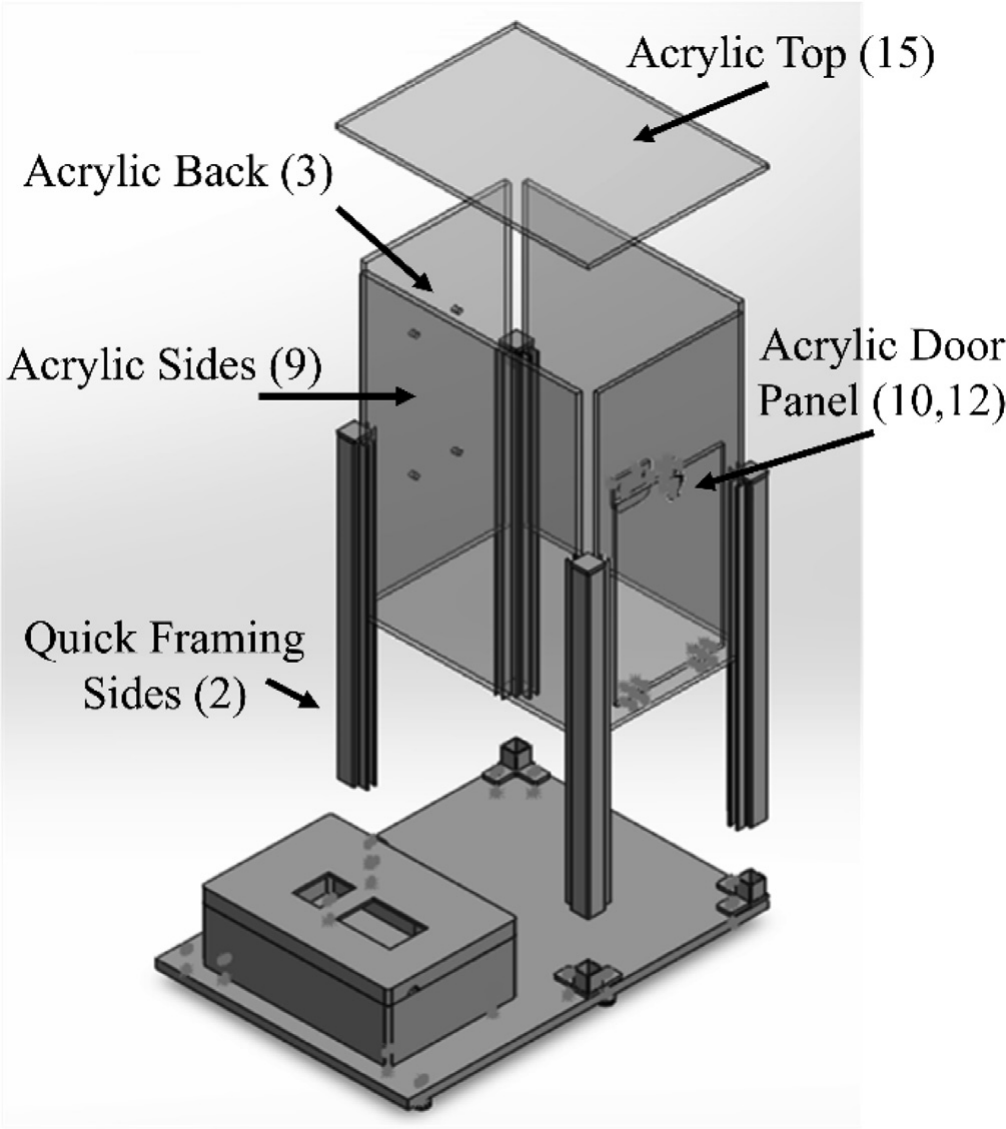
Assembly view of the machine. The framing is first attached, acrylic panels are slide on the sides, and the acrylic top is attached with Velcro.

**Fig. 15:**
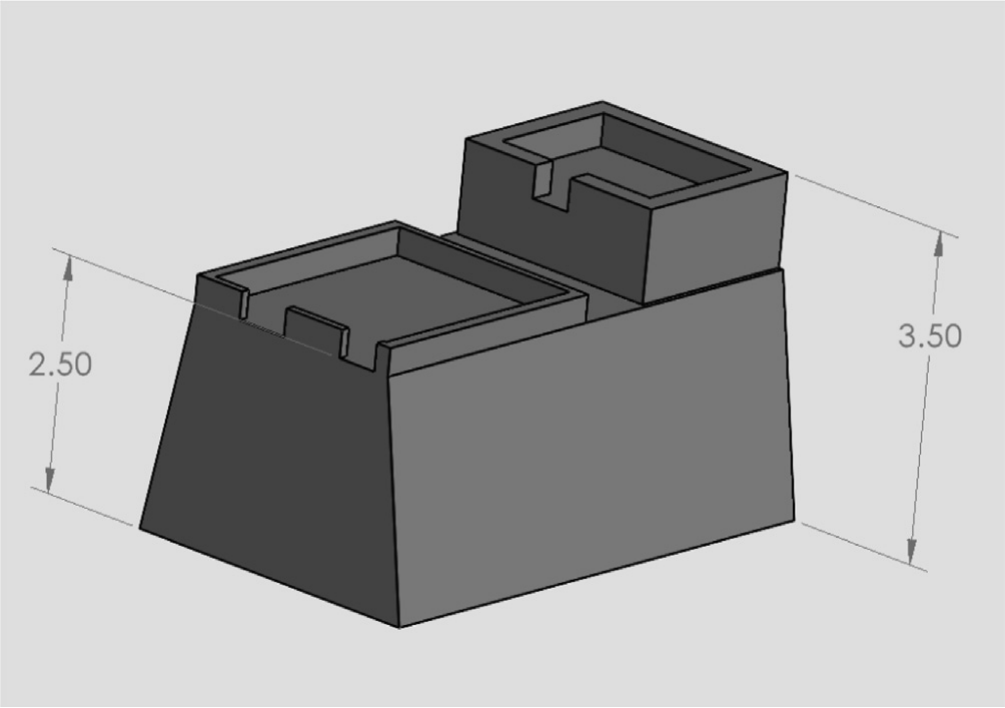
3D printable riser for lifting the touchscreen and reset button. This could be replaced with anything that will lift the screen to the top of the circuits box.
36. Place the ELEGOO UNO R3 and reset button on the riser.
37. Place the TB6600 motor driver and box portion of the power supply on each side of the riser holding the touch screen and reset button. This is shown in Fig. 16.

**Fig. 16:**
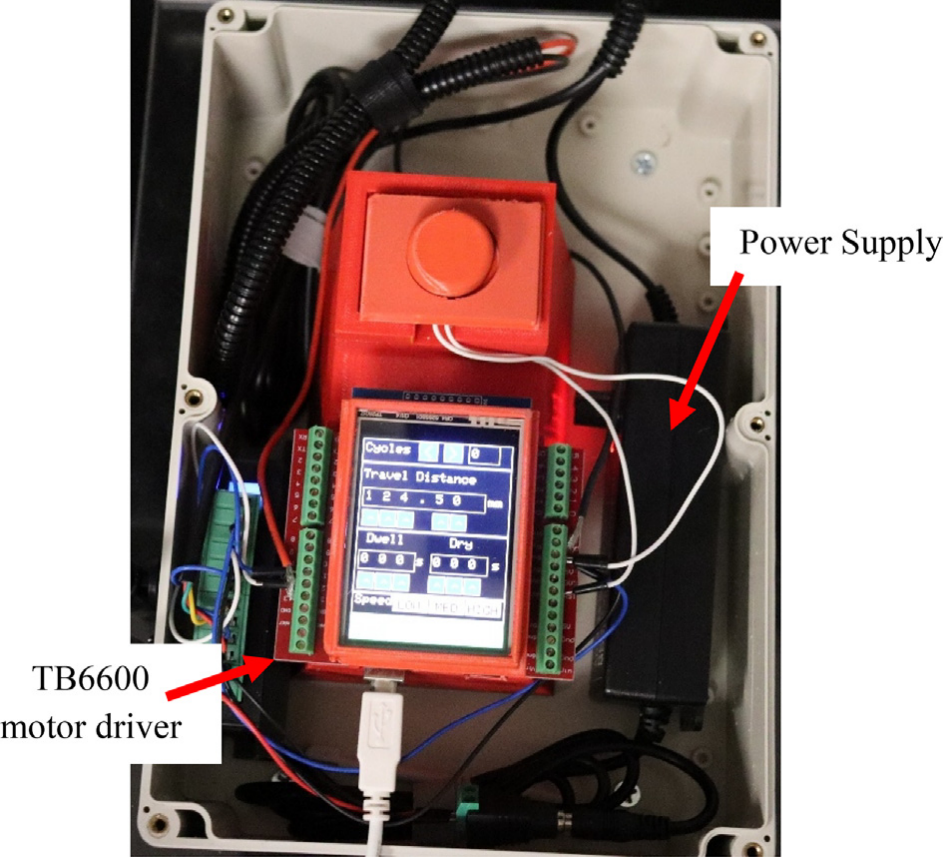
Completed circuit.
38. The USB wire comes out of the front of the box while the power cord, and cords that connect to the motor and limit switch go out of the back of the circuit box.
39. Place the lid of the circuit box over the circuit box, ensuring everything lines up with the holes that were cut. (Touchscreen, reset button, USB cord, wire loom tubing, and power supply cord.) Then, screw the lid on with the screws that came with the circuit box (Fig. 17).

**Fig. 17:**
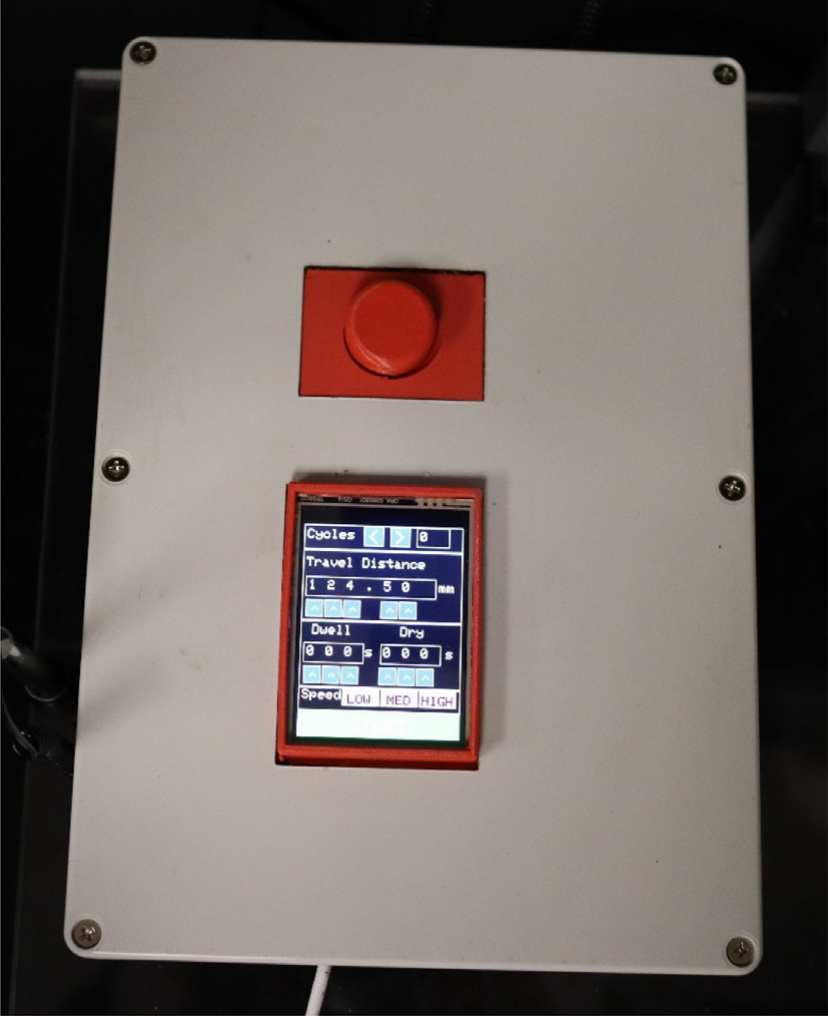
Outside of completed circuit box.
40. Put the caps of the quick framing on each framing piece.
41. Stick the Velcro on each of the four framing caps and in the four corners of the top piece of acrylic (15).
42. Attach the acrylic top to the machine (15).
43. Stick stylus on circuit box.
44. Cut the U channel edge trim into 4 pieces of approximately 4 in. long. Cut a 90- degree corner out of the top part of each piece in the center. After cutting the U channel edge bend it to create the shape shown in Fig. 18. Then glue each one onto the 4 top corners of the machine. These are included as a safety feature to avoid sharp corners around the top of the device.

**Fig. 18:**
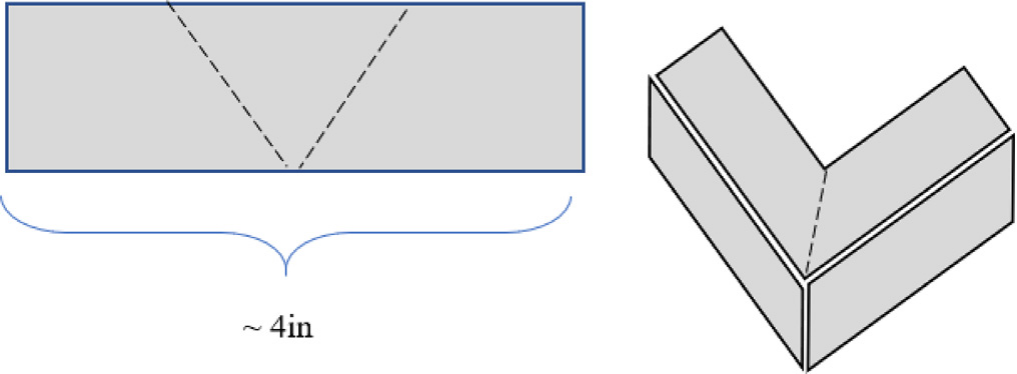
Diagram showing how to cut U channel and how to fold it to glue around 4 corners of machine top.

## 6 Operation instructions

### 6.1 Operating instructions – with preprogrammed variables

1. Plug the machine into a wall outlet.
2. Connect the USB cord to another power supply. (Computer or a USB wall charger.)
3. The machine will automatically turn on when plugged in.
4. Before it can be used, press the home button on the touch screen. This will move the mount of the dip coater up to the limit switch so the machine will start from the same height each time.
5. Clip the substrate to be coated to the mount of the machine.
6. Place a beaker of the coating in the machine under the substrate.
7. Now using the stylus, the desired settings can be chosen. There are 5 different variables that can be adjusted from the touchscreen.
  a. Cycles (variable c; default 1): This is the number of dips the machine will perform when start is pressed. One cycle is defined as going down the desired travel distance, dwelling for desired time and returning to the original starting height then holding for the desired drying time.
  b. Travel Distance (variable td; default 100 mm): This is how far down the mount will travel when start is pressed.
  c. Dwelling time (variable holddwell; default 0 s): This is the amount of time the mount will remain stopped once it’s traveled the complete travel distance down.
  d. Drying time (variable holddry; default 0 s): This is the amount of time the mount will remain stopped after returning to its starting position.
  e. Speed (variable speedVal; default 1250): This variable is related to the speed the mount will travel going up and down. The relationship between speedVal and the speed (mm/s) is $\mathrm{speedVal}=\frac{1250}{\mathrm{speed}}$. There are three speed settings programmed for the machine (low = 0.5 mm/s, med = 1 mm/s, fast = 5 mm/s). These can be adjusted by altering the code, specifically variable speedVal, and reuploading it to the ELEGOO UNO *R*3.
8. Once the desired settings are chosen, press start to start the cycles.
9. If the machine needs to be stopped at any point, press the reset button. This will reset the ELEGOO UNO R3 and the machine will stop moving. Once this is pressed, the touch screen will reset so home will need to be pressed again and the settings will need to be chosen again to continue operating the machine.

### 6.2 Operating instructions – editing code

1. Download Arduino IDE on a computer [4].
2. Connect the USB cord from the ELGOO UNO into the computer.
3. Open the “Dip Coater Code” file in Arduino IDE.
4. Change the desired features. Everything from the look of the touchscreen, max travel distance, speed values, etc., can be adjusted through the code.
5. The code is organized in the form:
  a. Installing libraries and Defining variables
    i. This section first imports the libraries needed for the touch screen. These libraries must be installed in Arduino IDE before using them.
    ii. It also defines the pins used for the touchscreen and motor connections.
    iii. Other variables are also defined here such as colors, initial values, travel distance, and the three speed options.
  b. Initial Setup
    i. This section of code is executed each time the machine is turned on. It includes details on which pins are outputs vs inputs and produces the initial display on the touch screen.
  c. Loop Function
    i. This section of code is executed continually after the machine is turned on.
    ii. The first thing that is done, is the travel distance, dwelling/ drying times are calculated. This is because the code is written such that in these cases, the place values are changed by the user, so these changes must be converted to variables to be used.
    iii. The beginning *if statement* checks if the touchscreen has been pressed.
    iv. Once the screen is pressed, the point is saved as coordinates and passed through a series of *if statements* to determine which button has been pressed.
      1. The first *if statement* is for the home button. When activated, the motor will move up to the limit switch and back down a few steps and stop. Then, the home button is replaced with a start button.
      2. The next *if statements* are for the cycle buttons. When pressed here, the number of cycles will increase/decrease by one.
      3. Then the travel distance buttons are listed. These will change the place value of the total travel distance.
      4. The next *if statements* control the speed. When activated, the selected speed is highlighted and the speed is set to the associated value.
      5. The dwelling time *if statements* are then listed followed by the drying time. These act similar to the travel distance by changing the place value of the times.
      6. Lastly, an *if statement* for the start button is listed. When this button is pressed, the machine will begin the dipping process, based on the selected speed, dwelling/ drying times, number of cycles, and total travel distance.
6. Any of the code can be adjusted to meet desired specifications. One helpful feature is the serial monitor in the Arduino IDE. It can be used to print out information to ensure your touchscreen is working properly without risking an issue with the motor. For example, if changing the location of the buttons on the screen is desired, a print line could be added after the if statement for the buttons. This print line would then output to the serial monitor each time the button is pressed.
7. The last step is to select the board “Arduino Uno” and the correct port. Then press the upload button.
8. Now the device is adjusted so that without the computer it will retain these changes.

## 7 Validation and characterization

**Fig. 19:**
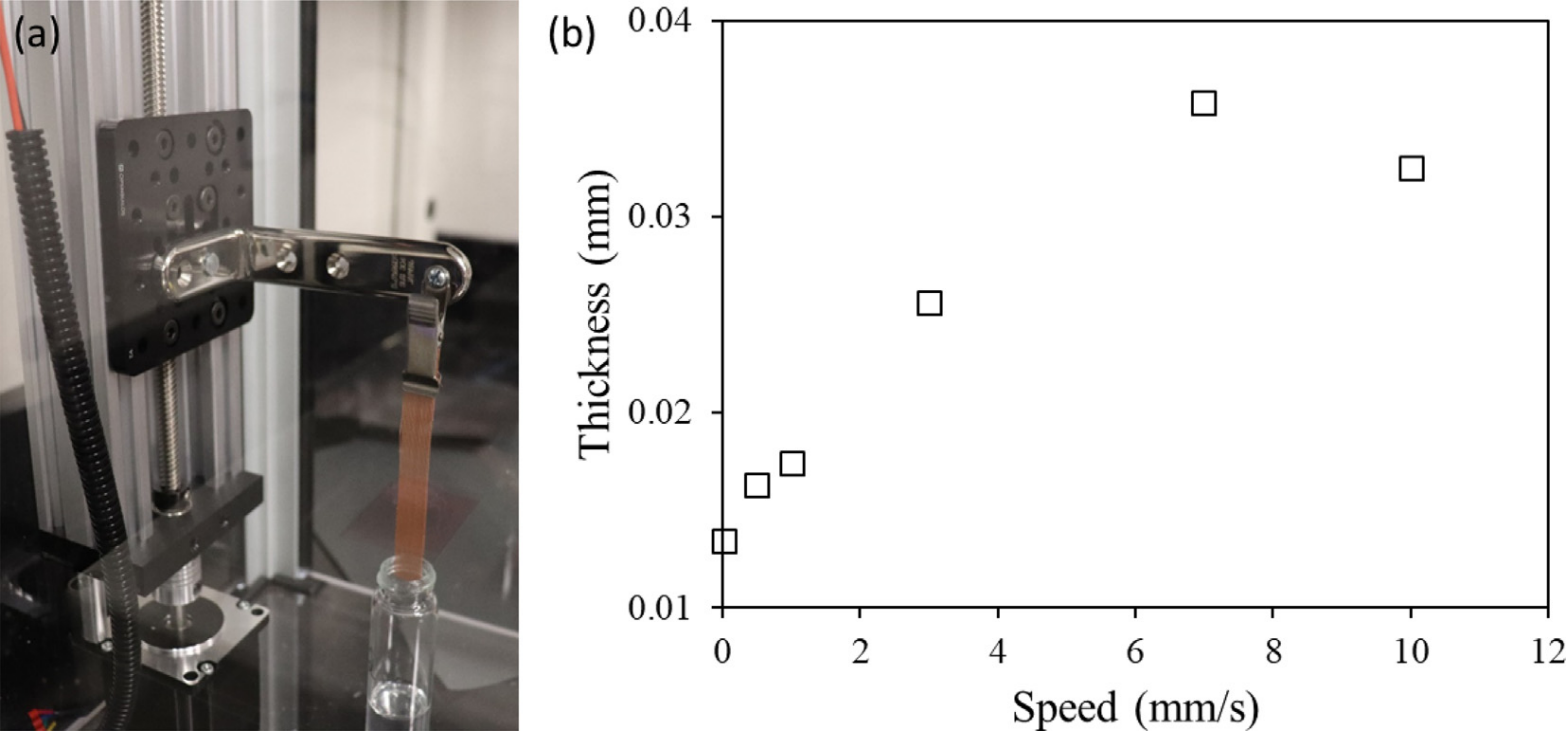
Dip coating test results showing (a) the dip coater in process of coating one copper mesh strip and (b) the thickness of coating versus the immersion/removal speed for the copper mesh after curing with UV light for 90 mins.

**Fig. 20:**
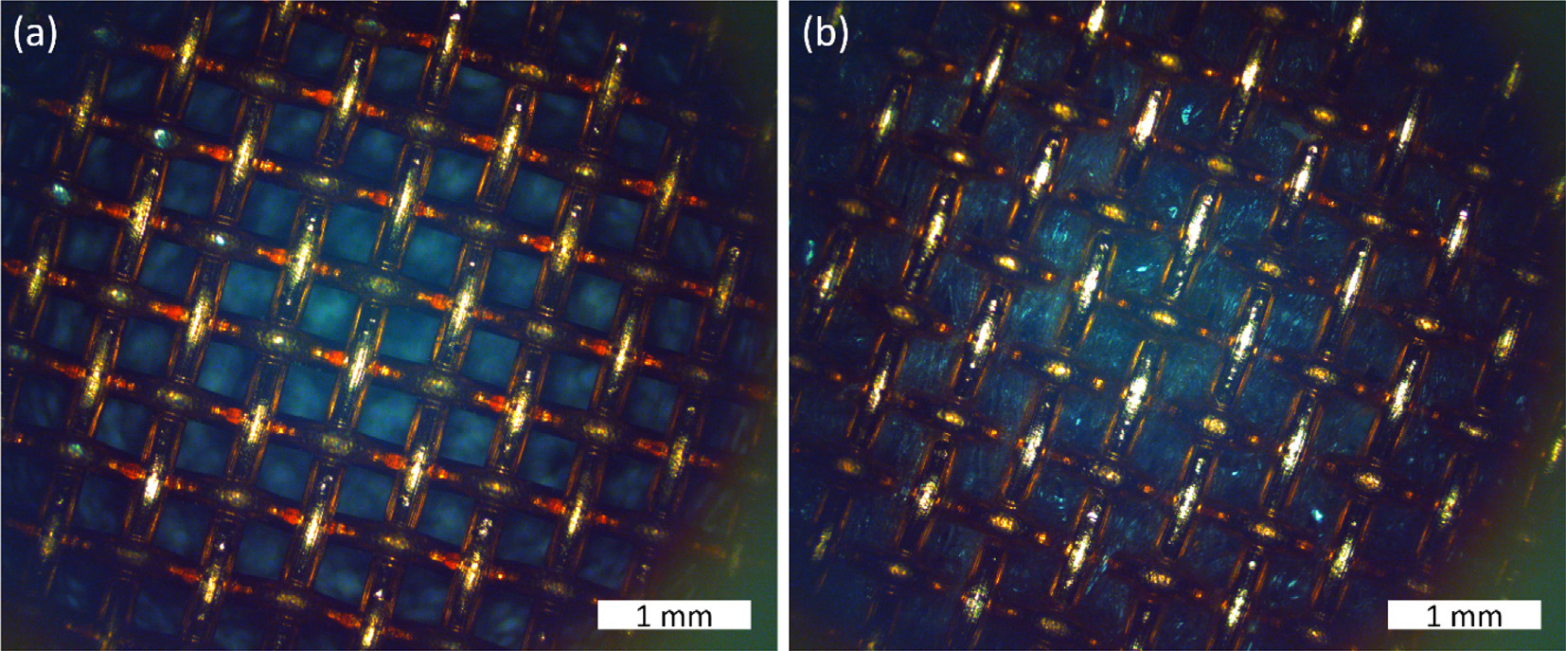
Microscopic images of copper mesh (a) before and (b) after coating with hydrogel, taken using a Navitar Zoom 6000 inspection microscope with a 5× objective.

To demonstrate the real-life applications of our dip-coater, two separate tests were performed. One to show the performance of the machine and another to show an application for the coating. Both tests use copper mesh and are coated in a PAM hydrogel made by mixing Acrylamide (AM), Methylenbisacrylamide (BIS), Polyacrylamide (PAM), 3′,5′-Diethoxyacetophenone (DEOP), and distilled water. [15], [16]. These ingredients were proportioned by weight at 50:1.5:0.5:1:47 wt% respectively. DEOP is a photoinitiator, where the polymerization is initiated by reactive species such as radicals [17]. It was found that an increase in the monomer concentration created the need for a longer time to mix. To adjust for this factor, the first three ingredients were mixed with the water for up to 1 h at 600 rpm. Because DEOP creates a photosensitive effect, this ingredient was added to the solution last and was mixed for about 30 more minutes. To test the performance of the dip coating machine, one experiment used a 0.009″ diameter copper mesh cut into 6 separate substrates. Each strip was dipped in the hydrogel solution at different speeds; 0.01, 0.5, 1, 3, 7 and 10 mm/s. Each run of the dip coater used 1 cycle and a 10 s dwell time. The width of each mesh strip was measured using a caliper and the mass of each sample was measured before coating with an analytical balance. After coating the mesh strips, they were cured under a UV light for 90–240 mins. After 90 min, each sample’s mass was once again recorded. Then, the length of the coating coverage was measured using a caliper. Using these measurements, the thickness of the coating for each speed after the curing process was calculated from $\mathrm{thickness}=\frac{m}{\rho lw\alpha }$. Where *m* is the mass of the coating, *ρ* is the density of the hydrogel, *l* is the length of the coating, and *w* is width of the copper foil. And $\alpha =\frac{2\pi D}{S}$ represents the ratio of the active surface area over footprint, where *D* is the diameter of the wire and *S* is the spacing between wire. The copper mesh used here has a diameter of 0.009″ and a spacing of 0.011″. The density was found by averaging the densities based on the percent weight of the ingredients in the mixture. $\rho_{\mathrm{mix}}=100/\left(\frac{50}{\rho_{\mathrm{AM}}}+\frac{1.5}{\rho_{\mathrm{BIS}}}+\frac{0.5}{\rho_{\mathrm{PAM}}}+\frac{1}{\rho_{\mathrm{DEOP}}}+\frac{47}{\rho_{\mathrm{water}}}\right)$. Fig. 19 shows these thicknesses compared to the set speed for the dip coater. As observed in Fig. 19, the thickness increases with the immersion/removal speed up to 6.98 mm/s and then get saturated. The post cure 10 mm/s sample thickness is lower than the 7 mm/s post cure trial. This could be attributed to error in the measurements or some of the mesh breaking off at some point during the process and effecting the mass measurement. Fig. 20 shows the microscopic images of the copper mesh before and after coating in the hydrogel, taken using Navitar Zoom 6000 optical microscope.

**Fig. 21:**
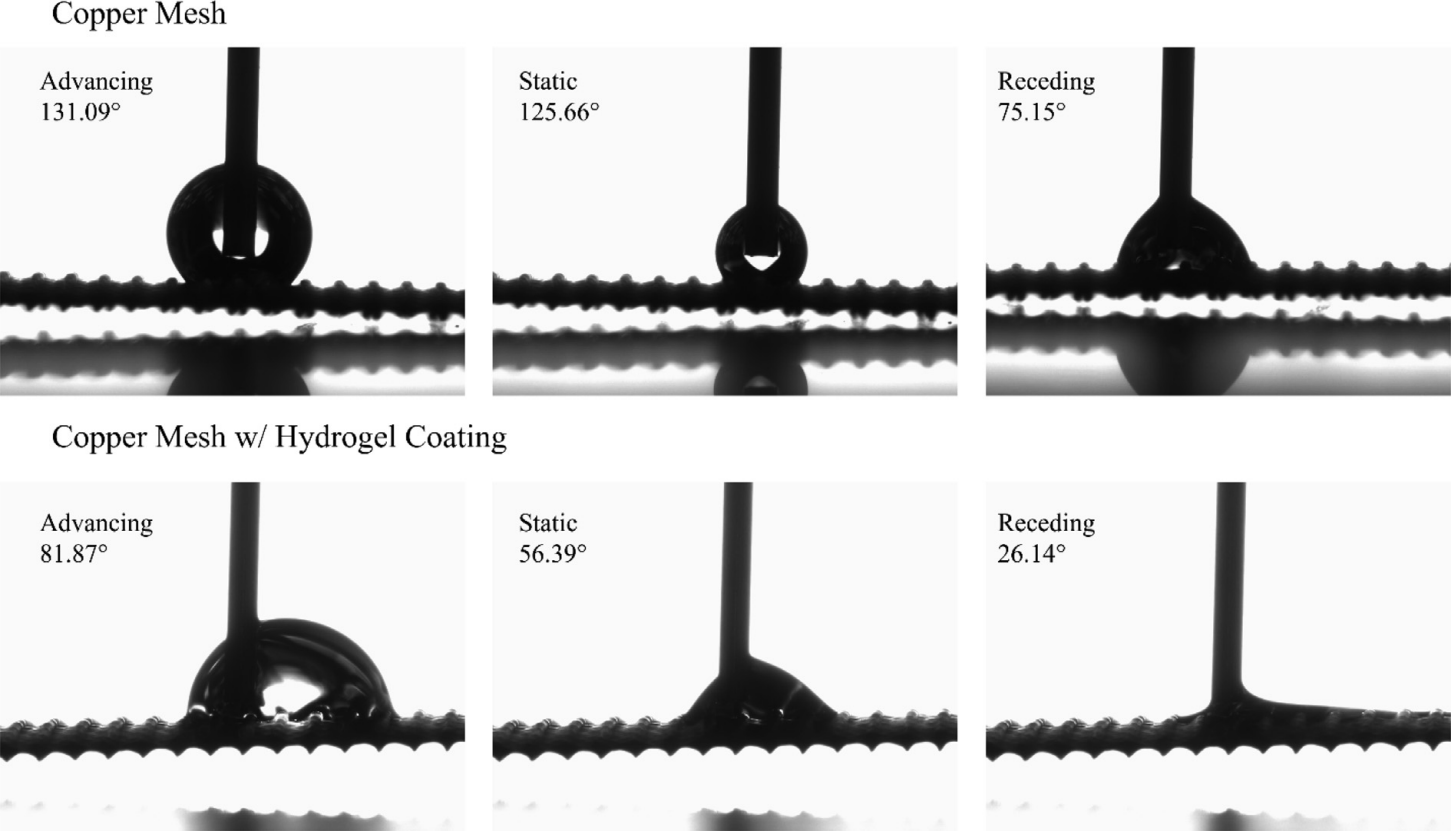
Contact angle measurements of copper meshes before and after coating with hydrogel using a goniometer with distilled water and a starting droplet of 2 μL and 15 μL/2s speed with a 6 s break.

**Fig. 22:**
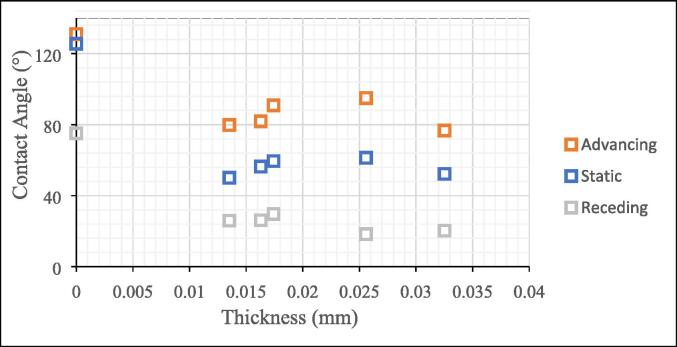
Static and dynamics contact angles of copper meshes with hydrogel coating versus the thickness of coating layers. Thickness of 0 represents copper mesh without coating.

This hydrogel coating makes the mesh superoleophobic and superhydrophilic with a water contact angle of less than 10 degrees. Fig. 21 shows the snapshots of water contact angle observed on one of the coated copper meshes, taken using OCA 15EC contact angle goniometer. Fig. 22 shows the effect of the coating thickness on the static, advancing, and receding contact angle changes. The contact angle is lower than the mesh with no coating so it did produce a hydrophilic effect but not to the expected extent. It can be seen that all three contact angles are higher than the copper mesh with any thickness of hydrogel applied. The coated mesh can be used to separate oil and water to assist in many different processes. This would include, but not be limited to, oil spills, manufacturing wastewater, and water produced in drilling and fracking processes. The other experiment used a 3″ × 3″ copper mesh with 0.0022″ diameter wire. The experiment began by adjusting the settings on the touch screen to have the machine perform 2 cycles with a travel distance of 130.00 mm, dwell time of 10 s, and dry time of 10 s. After automatic calibration, the machine was able to perform the work that it was set to do smoothly with no significant issue. The sample was then removed from the machine and moved to a UV resin curing light chamber to dry out for 4 h. The sample should be able to isolate water from oil after this dipping and curing process. The result from this test run showed that the actual performance and the predicted performance are on par with each other which means that the project was executed successfully.

## Declaration of Competing Interest

The authors declare that they have no known competing financial interests or personal relationships that could have appeared to influence the work reported in this paper.

## Figures and Tables

**Design files from other sites needed in complete assembly:** 

Type	Design File		Link
Actuator Assembly	C-Beam-Linear Actuator 250 mm	OpenBuilds	https://grabcad.com/library/c-beam-linear-actuator-250mm-1
	Corner Bracket	McMaster-Carr	https://www.mcmaster.com/15655A41/
	Spring Clamp	McMaster-Carr	https://www.mcmaster.com/6076A17/

Machine Framing Files	2115 Panel Gasket	80/20	https://8020.net/2115.html
	4483 Door Stop	80/20	https://8020.net/4483.html
	6082A12_DRAW LATCH	McMaster-Carr	https://www.mcmaster.com/6082A12/
	Corner Base Connector		https://8020.net/9242.html
	End Cap		https://8020.net/9210.html
	Quick Frame Corner		https://8020.net/9030.html
	Hinge	McMaster-Carr	https://www.mcmaster.com/1598A52/

Screws	8.8 Steel Hex Screw 16 mm	McMaster-Carr	https://www.mcmaster.com/91280A136/
	Swivel Leveling mount with threaded hole	McMaster-Carr	https://www.mcmaster.com/6103K81/
	Hex nut for actuator (M4x0.7 mm) (90591A255)	McMaster-Carr	https://www.mcmaster.com/catalog/128/3500
	Hex nut for Phillips screw (larger) (90480A195)	McMaster-Carr	https://www.mcmaster.com/catalog/128/3498
	Hex nut for Phillips screw (smaller) (90480A005)		https://www.mcmaster.com/catalog/128/3498
	Hillman 10-32x34 in Phillips screw (med) (91735A831)	McMaster-Carr	https://www.mcmaster.com/catalog/128/3308
	Low Profile Screw M5 (0.8 mm)	OpenBuilds	https://openbuildspartstore.com/low-profile-screws-m5-10-pack/
	Phillips screw (large) (91735A833)	McMaster-Carr	https://www.mcmaster.com/catalog/128/3308
	Phillips screw (small) (91735A106)	McMaster-Carr	https://www.mcmaster.com/catalog/128/3308
	Smaller Phillips screw	McMaster-Carr	https://www.mcmaster.com/sheet-metal-screws/steel-phillips-rounded-head-screws-for-sheet-metal/
